# Study on the Metabolic Basis of the Color Formation of Two Color-Presenting Types of Jujube Fruits

**DOI:** 10.3390/foods13172657

**Published:** 2024-08-23

**Authors:** Xiaofeng Zhou, Qianqian Shi, Xingang Li, Ze Yuan, Min Yan, Dengyang Lu, Yan Wang, Xiaoqiu Pu, Cuiyun Wu

**Affiliations:** 1 College of Life Science and Technology, Tarim University, Alar 843300, China; zhxf112233@163.com (X.Z.);; 2 College of Forestry, North West Agriculture and Forestry University, Xianyang 712100, China; 3 College of Horticulture and Forestry, Tarim University, Alar 843300, China

**Keywords:** jujube, pigment, carotenoid, anthocyanidin, metabolome

## Abstract

Jujube is a plant of the genus Ziziphus in the family Rhamnaceae; its fruit has high nutritional value, and it is rich in polyphenols, flavonoids, and other secondary metabolites. The color of its peel is an important indicator for evaluating the appearance of the fruit. However, the mechanism of the difference in color presentation between the seedling offspring of the ‘Red Fruit’ (TLHH) and the ‘Green Fruit’ (TLHL) of the fresh jujube cultivar ‘Tailihong’ is not clear. Therefore, this study used targeted metabolomics techniques to accurately and quantitatively analyze the metabolic pathways of carotenoid and anthocyanin metabolites during the ripening process of two color-presenting types of jujube fruits. Through the analysis of the dynamic changes in the pigment content of the jujube peel, it was found that 30 DAP (days after pollination), 80 DAP, and 110 DAP were the key periods for the development of the color of the peel of ‘TLHL’ and ‘TLHH’ jujube and that the substances responsible for the main differences were chlorophyll, carotenoids, and anthocyanins. Furthermore, we used an LC-MS/MS metabolic analysis to compare the differences in the carotenoids and anthocyanin metabolites between the two color-presenting types of jujube peels at the key periods of 30 DAP, 80 DAP, and 110 DAP. We detected 32 carotene metabolites and 75 anthocyanin metabolites, respectively, among which lutein had the highest content of carotenoids; it reached the maximum value (93.05 µg/g) and was higher than that of ‘TLHH’ (74.14 µg/g) at 30 DAP of ‘TLHL’. Both showed a decreasing trend with fruit ripening. The anthocyanin with the highest content was cyanidin-3-O-(tartaryl)rhamnoside-5-O-glucoside, which reached the maximum value (258.32 µg/g) at 30 DAP of ‘TLHH’ and was 51.6 times that of ‘TLHL’; similarly, both showed a decreasing trend with fruit ripening. These results elucidate the main metabolites of carotenoids and anthocyanins in the two types of jujube peel and their accumulation characteristics, suggesting that the key metabolites of the difference in color between ‘TLHL’ and ‘TLHH’ jujube fruits were lutein and cyanidin-3-O-(tartaryl)rhamnoside-5-O-glucoside, increasing the understanding of the color mechanism of jujube peel and providing a reference for targeted genetic breeding of jujube peel color.

## 1. Introduction

The Chinese jujube (*Ziziphus jujuba* Mill.) is one of the most widely cultivated and economically important tree species in the family Rhamnaceae. Jujube fruits contain polyphenolic substances, such as phenolic acids, flavonoids, flavanols, and anthocyanins [1,2,3], which not only have high nutritional value [4] but also play an important role in fruit coloration [5,6]. Jujube fruit color is an important appearance index that is used to measure the comprehensive quality and grading of the fruit. In recent years, the research on jujube fruit coloring and peel pigmentation substances has mainly focused on the main cultivars of the ‘Dongzao’ jujube and ‘Junzao’ jujube. Zhang’s [7] studies demonstrated that with the coloring of the ‘Dongzao’ jujube fruit, the contents of chlorophyll, total flavonoids, total carotenoids, and total saponins in the peel decreased significantly. However, in Li’s [8] research results on the ‘Dongzao’ jujube peel, carotenoid content had been decreasing significantly throughout the developmental process, and the content of proanthocyanidins increased significantly and then decreased significantly, with the highest content occurring at the semi-red stage. The chlorophyll a, chlorophyll b, and β-carotene contents of ‘Junzao’ jujube peel had been decreasing throughout the developmental process; as a whole, the total phenolics, total flavonoids, and total flavanols showed an overall increase and then decrease, and the lutein content decreased sharply before the first red stage and then increased; this pattern of change suggests that lutein seems to be associated with the formation of the color in the jujube peel at the fully ripened stage [9].

Different jujube varieties have different pigment types and components for fruit coloring [9,10,11]. Five flavonols were detected in the peel of ripe ‘Dongzao’ jujube: quercetin derivative, quercetin 3-robinobioside, quercetin 3-rutinoside, quercetin derivative, and vderivative, and eight flavanols were detected: procyanidin B4, catechin tripolymer, catechin, procyanidin B1, catechin tripolymer, epcatechin, catechin tripolymer, and catechin dimer; no anthocyanins were detected [10]. The flavanols were synthesized in large quantities when the fruits appeared green, and their content decreased as the fruits turned red. However, the flavonols had the highest content when the fruits were fully red [10]; of these, quercetin accumulated in large quantities in the course of the process from white ripening to fully red, promoting the red transformation [11]. Further studies found that three anthocyanins (delphinidin, delphinidin 3-O glucoside, and dimethyl delphinidin 3-O glucoside), three flavonols (isorhamnetin O-hexoside, isorhamnetin 5-O hexoside, and isorhamnetin 3-O glucoside), and three flavonoids (luteolin, C-hexosyl-luteolin C-pentososide, and 8-C-hexoside-luteolin O-hexoside) were accumulated in the peel [7]; the content of flavonoid glycosides increased steadily with the deepening of the coloring degree of the peel [12], while the proanthocyanidins and the flavanols catechin and epicatechin were significantly reduced [13]. Sixteen phenolic compounds were isolated from the peel of ‘Junzao’ jujube by HPLC, including six flavanols (proanthocyanidins B1, proanthocyanidins B2, proanthocyanidins B3, quercetin, (−) epicatechin, and (+) catechins), four flavonols (quercetin-3-galactoside, quercetin-3-glucoside, quercetin-3-rutinoside, and quercetin-3-rhamnoside), and six phenolic acids (gallic acid, chlorogenic acid, caffeic acid, ferulic acid, and cinnamic acid). Among them, quercetin glycosides were the main flavonol substances [9], and the content of quercetin 3-rhamnoside and quercetin 3-rutin (rutin) was higher in the peel [14].

In the seedling selection population constructed by our group in the previous breeding, we found two individual plants ‘Green Fruit’ (TLHL) and ‘Red Fruit’ (TLHH) with different color variations of jujube fruits, where ‘TLHL’ fruit color varied as: green (30 DAP), cyan (50 DAP), yellow-white (80 DAP), red/white (95 DAP), and red (110 DAP); ‘TLHH’ showed: purplish red (30 DAP), pink (50 DAP), pinkish white (80 DAP), orange/white (95 DAP), and red (110 DAP). In order to explore the special color-presenting mechanism of its fruits, this study took the ‘TLHL’ jujube fruit as the control and carried out a study on the differences in the color changes in the two types of jujube fruits. Through the measurement of physiological indexes and metabolomics analysis, we compared the main differential metabolites and their accumulation characteristics between the two types of jujube peels; this provided a reference with which to elucidate the biological basis of the color formation of the two types of jujube peels.

## 2. Materials and Methods

### 2.1. Plant Materials

This study was conducted in the Jujube Germplasm Resource Nursery of Tarim University, Alar, Xinjiang. This resource nursery is located at the latitude of 40°32′ N and longitude of 81°17′ E, with an average altitude of 1100 m. The temperature difference between day and night is large (16 °C–31 °C) (https://weather.cma.cn/web/weather/51730.html (accessed on 10 October 2022)), precipitation is scarce, and light hours are long. The average annual temperature is 10.7 °C, the accumulated temperature of ≥10 °C is 4113 °C, the frost-free period is 220 days, the average annual sunshine hours are 2556.3~2991.8 h, the average annual precipitation is 40.1~82.5 mm, and the soil is light salinized soil.

The fruits of the seedling offspring ‘Green Fruit’ (‘TLHL’ for short) and the seedling offspring ‘Red Fruit’ (‘TLHH’ for short) of the ‘Tailihong’ jujube were collected from the Jujube Germplasm Resource Nursery of Tarim University. The peel of the fruit was sampled at 30, 50, 80, 95, and 110 days after pollination (DAP), respectively. Thirty fruits of uniform size, free of pests and diseases, on biennial branches in different directions on the periphery of the canopy were randomly selected from each sample tree; each sample comprised three biological replicates, for a total of ninety fruits. Immediately after picking, the samples were put into a low-temperature sampling box and brought back to the laboratory in a timely manner; the peel and pulp were separated, and the peel was quick-frozen with liquid nitrogen and transferred to an ultra-low temperature refrigerator at −80 °C to be measured.

### 2.2. Instruments

Ultrapure water instrument UPT-1-107, Youpu Company, Sichuan, China; grinder MM400, Retsch Instrument Company, Düsseldorf, Germany; electronic balance FA 1104N, Shanghai Pohai Instrument Company, Shanghai, China; refrigerated centrifuge H2100R, Xiangyi Instrument Company, Xiangtan, China; pipette gun, Eppendorf AG, Hamburg, Germany.

### 2.3. Determination of Physiological Indicators

#### 2.3.1. Determination of Color Difference Index

A handheld CR-410 colorimeter was used to conduct measurements at the equator on the shaded and sunny sides of the fruit. The equipment was programmed for two flashes per sample. Light source: C light source (balanced light); observation angle: 2°. A positive value of ‘a’ indicates a reddish coloration, while a negative value of ‘a’ indicates a greenish coloration. A positive value of ‘b’ indicate a yellowish color, while a negative value of ‘b’ indicate a bluish color.

#### 2.3.2. Extraction and Determination of Chlorophyll and Carotenoids

The acetone method was used [15] as follows: weigh 0.1 g of jujube peel, add 1.0 mL of 80% acetone to it, and grind it fully using a grinder; conduct ultrasonication after grinding at 300 W for 30 min; and transfer the homogenate to a refrigerator at 4 °C for 24 h and centrifuge it at 12,000 rpm for 10 min. Eighty percent acetone was used as a blank control, and the colors were measured at 470 nm, 646 nm, 652 nm, and 663 nm using a microplate reader.

The content of chlorophyll and carotenoids in the sample was calculated by the following formula.

C_a_ = 12.21A_663_ − 2.81A_646_

C_b_ = 20.13A_646_ − 5.03A_663_

Chlorophyll content = 1000A_652_/34.5

Carotenoid content = (1000A_470_ − 3.72C_a_ − 104C_b_)/229

where: C_a_—chlorophyll a concentration (μg/mL);

C_b_—chlorophyll b concentration (μg/mL);

Vt—total volume of extract (mL);

M—sample mass (g).

#### 2.3.3. Extraction and Determination of Total Phenols, Flavonoids, and Proanthocyanidins

About 0.05 g of jujube powder sample was weighed accurately; to this, 1.5 mL of 80% methanol solution was added and ground fully; after grinding, ultrasonication for 30 min and centrifugation at 12,000 rpm for 10 min were performed [16]. The test solution of the peel was obtained and stored in the refrigerator at −4 °C for later use. The total phenol content was determined using the forintol reagent method [16], with gallic acid as a standard. The total flavonoid content was determined using the aluminum nitrate-sodium nitrite colorimetric method [17], with rutin as a standard. The proanthocyanidin content was determined using the vanillin reagent method [8], with catechin as a standard (Table A1).

#### 2.3.4. Extraction and Determination of Anthocyanins

A total of 0.1 g of jujube peel was accurately weighed; to this, 1.0 mL of 0.1% hydrochloric acid methanol solution was added; then, it was fully milled. After milling and ultrasonication for 30 min, the homogenate was transferred to a refrigerator at 4 °C for 2 d; the supernatants were collected after being centrifuged at 12,000 rpm for 10 min, and the supernatant was to be tested. A methanol solution of 0.1% hydrochloric acid was used as a blank control, and the colorimetric conditions were defined as 530 nm and 657 nm. The experiment was repeated three times; the average value was obtained, and the following formula was used to calculate the anthocyanin content of the jujube peel: anthocyanin content = (A_530_ − 0.25 × A_657_)/sample mass [18].

### 2.4. Determination of Carotenoid Metabolites

#### 2.4.1. Preparation and Extraction of Samples

The jujube peels were freeze-dried, ground into powder (30 Hz, 1.5 min), and stored at −80 °C in an ultra-low temperature refrigerator. Fifty milligrams of Mg powder was weighed and extracted with 0.5 mL of a mixed solution of n-hexane/acetone/ethanol (1:1:1, *v*/*v*/*v*). The extract was vortexed for 20 min at room temperature. The supernatants were collected after being centrifuged at 12,000 r/min for 5 min at 4 °C. The residue was re-extracted by repeating the above steps under the same conditions; then, it was evaporated to dryness and reconstituted in a mixed solution of MeOH/MTBE (1:1, *v*/*v*). The solution was filtered through a 0.22 μm membrane filter for further LC-MS/MS analysis [19].

#### 2.4.2. UPLC Conditions

The carotenoid contents were detected using MetWare V 3.0 (http://www.metware.cn/) based on the UPLC-APCI-MS/MS system (UPLC, ExionLC™ AD, https://sciex.com.cn/; MS, Applied Biosystems 6500 Triple Quadrupole, https://sciex.com.cn/). The analytical conditions were as follows: LC: column, YMC C30 (3 μm, 100 mm × 2.0 mm i.d); solvent system: methanol/acetonitrile (1:3, *v*/*v*) with 0.01% BHT and 0.1% formic acid (A), methyl tert-butyl ether with 0.01% BHT (B); gradient program: started at 0% B (0–3 min), increased to 70% B (3–5 min), then increased to 95% B (5–9 min), and finally ramped back to 0% B (10–11 min); flow rate: 0.8 mL/min; temperature: 28 °C; injection volume: 2 μL. The APCI source operation parameters were as follows: ion source: APCI+; source temperature: 350 °C; curtain gas: set at 25.0 psi. The carotenoids were analyzed using scheduled multiple reaction monitoring. Data acquisitions were performed using Analyst 1.6.3 software (Sciex). Multiquant 3.0.3 software (Sciex) was used to quantify all metabolites. The mass spectrometer parameters, including the declustering potentials and collision energies for the individual multiple reaction monitoring transitions, were conducted with further declustering potentials and collision energies optimization. A specific set of multiple reaction monitoring transitions were monitored for each period according to the metabolites eluted within this period [20,21].

### 2.5. Determination of Anthocyanin Metabolites

#### 2.5.1. Sample Preparation and Extraction

The sample was freeze-dried, ground into powder (30 Hz, 1.5 min), and stored at −80 °C until needed. Fifty milligrams of powder was weighted and extracted with 0.5 mL of methanol/water/hydrochloric acid (500:500:1, *v*/*v*/*v*). Then, the extract was vortexed for 5 min; ultrasonication for 5 min and centrifugation at 12,000× *g* under 4 °C for 3 min were performed. The residue was re-extracted by repeating the above steps under the same conditions. The supernatants were collected and filtrated through a membrane filter (0.22 μm, Anpel, Shanghai, China) before LC-MS/MS analysis [22].

#### 2.5.2. UPLC Conditions

The anthocyanin contents were detected using MetWare V 3.0 (http://www.metware.cn/) based on the UPLC-ESI-MS/MS system. The analytical conditions were as follows: UPLC: column: WatersACQUITY BEH C18 (1.7 µm, 2.1 mm × 100 mm); solvent system: water (0.1% formic acid)/methanol (0.1% formic acid); gradient program: 95:5 *v*/*v* at 0 min, 50:50 *v*/*v* at 6 min, 5:95 *v*/*v* at 12 min, hold for 2 min, 95:5 *v*/*v* at 14 min, hold for 2 min; flow rate: 0.35 mL/min; temperature: 40 °C; injection volume: 2 μL. The ESI source operation parameters were as follows: ion source: ESI+; source temperature: 550 °C; ion spray voltage: 5500 V; and curtain gas: set at 35 psi, respectively [23]. The anthocyanin metabolites were qualitatively and quantitatively analyzed in the same ways as the carotenoid metabolism.

### 2.6. Statistical Analysis

Each experiment had three biological replicates. The *t*-test statistical method was used for data analysis. GraphPad Prism 9.0 software (GraphPad Software, Santiago, CA, USA) was used for the cluster analysis and plotting of independent samples. Origin 2021 (OriginLab Corporation, Northampton, MA, USA) was used for a PCA analysis. The discriminant method of differential metabolites was the relative content of metabolites in fold change ≥ 2 and fold change ≤ 0.5, which was considered a significant difference in metabolites. MetaboAnalyst Perl 5.28.1 was used for a pathway analysis.

## 3. Results

### 3.1. Comparison of Color Changes during the Development of Jujube Fruits

**Figure 1:**
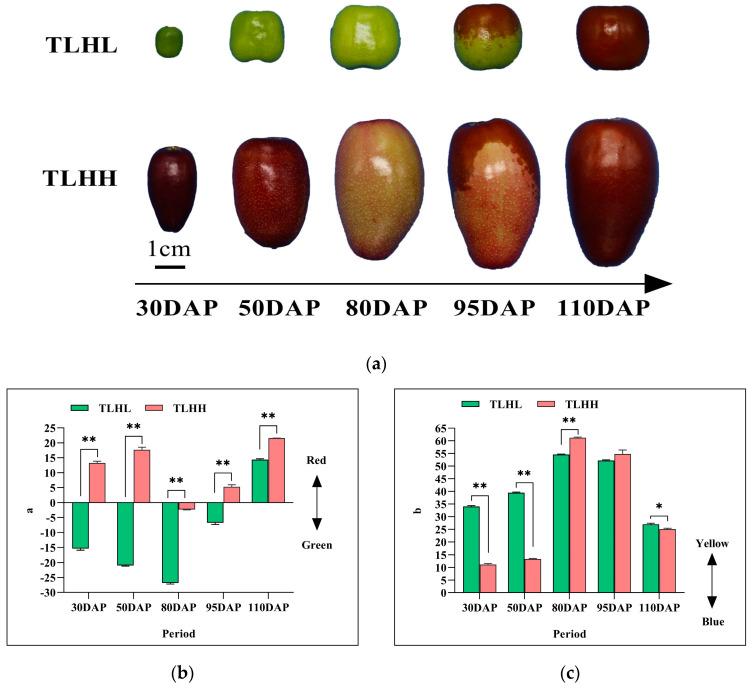
Changes in peel color difference index during jujube development. (**a**) Phenotypes of ‘TLHL’ and ‘TLHH’ jujube fruits at five developmental stages; (**b**,**c**) changes in the color difference index of jujube peel. Values of three replicates are expressed as the means ± SD. * indicates a significant difference (*p* < 0.05), and ** indicates a very significant difference (*p* < 0.01) by *t*-test, the same below.

As can be seen in Figure 1, there is a difference in the color changes in the ‘TLHL’ and ‘TLHH’ fruits during development (Figure 1a). A positive value of ‘a’ indicates a reddish coloration, while a negative value of ‘a’ indicates a greenish coloration. With the exception of 80 DAP, the ‘a’ values of the ‘TLHH’ fruit in all periods were positive. The ‘TLHL’ fruit ‘a’ was negative before 95 DAP and positive when the peel was red at 110 DAP (Figure 1b). Positive values of ‘b’ indicate a yellowish color, while negative values of ‘b’ indicate a bluish color; the ‘b’ values of both the ‘TLHL’ and the ‘TLHH’ peels were positive (Figure 1c). With the ripening of the fruits, the ‘b’ values of both the ‘TLHL’ and the ‘TLHH’ jujube peels showed a ‘single peak’ trend, reaching a maximum at 80 DAP, and ‘TLHH’ was significantly higher than ‘TLHL’.

### 3.2. Analysis of the Dynamics of Peel Pigment Content in ‘TLHL’ and ‘TLHH’ Jujube Fruits

**Figure 2:**
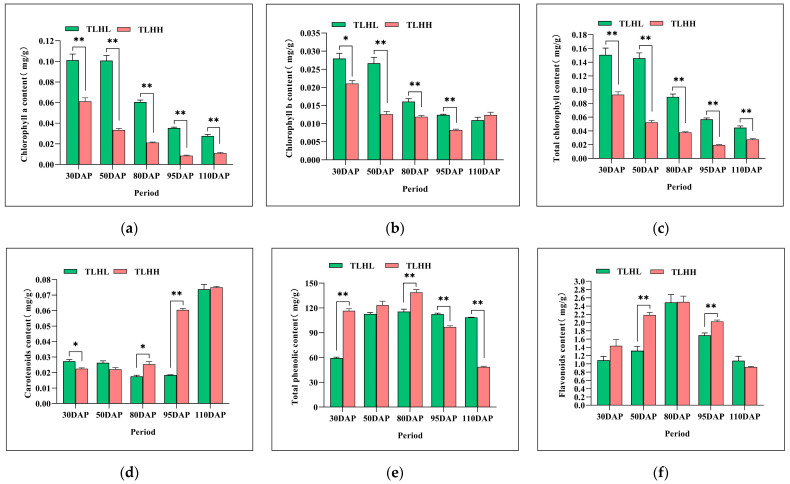

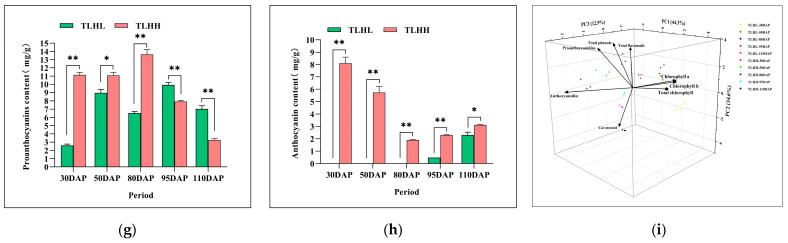
Changes in peel pigment content and principal component analysis during jujube development. (**a**–**c**) Changes in the contents of chlorophyll a, chlorophyll b, and total chlorophyll in the jujube peel, respectively; (**d**) changes in carotenoid content in the jujube peel; (**e**) changes in flavonoid content in the jujube peel; (**f**) changes in the content of total phenols in the jujube peel; (**g**) changes in the content of proanthocyanidins the jujube peel; (**h**) changes in the content of anthocyanins in the jujube peel; (**i**) principal component analysis of pigment content in the jujube peel. * indicates a significant difference (*p* < 0.05), and ** indicates a very significant difference (*p* < 0.01) by *t*-test.

As can be seen in Figure 2, the contents of chlorophyll a, chlorophyll b, and total chlorophyll in the peels of ‘TLHL’ and ‘TLHH’ decreased during the development process (Figure 2a–c), and the contents of chlorophyll a and total chlorophyll in the peel of ‘TLHL’ were always significantly higher than those of ‘TLHH’; with the exception of the level at 110 DAP, the content of chlorophyll b in the peel of ‘TLHL’ was significantly higher than that of ‘TLHH’ in all the periods. The trend of the carotenoid content in the peel of the ‘TLHL’ and ‘TLHH’ jujube was opposite to that of the chlorophyll (Figure 2d). The carotenoid content of the ‘TLHL’ jujube peel was higher than that of ‘TLHH’ at 30 DAP and 50 DAP; the carotenoid content of the ‘TLHL’ jujube peel continued to decrease; the carotenoid content of the ‘TLHH’ jujube peel continued to increase at 80 DAP; and the carotenoid content of the ‘TLHH’ jujube peel was significantly higher than that of ‘TLHL’ at 95 DAP. The carotenoid contents of both the ‘TLHL’ and the ‘TLHH’ jujube peels increased rapidly to over 0.07 mg/g at 110 DAP. The contents of total phenols and flavonoids in the peels of both the ‘TLHL’ and the ‘TLHH’ jujube showed an increasing and then decreasing trend (Figure 2e,f), reaching the highest level at 80 DAP; the total phenol content of the ‘TLHH’ jujube peels was significantly higher than that of ‘TLHL’, and the contents of the total phenols and flavonoids in the peel of the ‘TLHH’ jujube decreased to the lowest level at the fully red stage at 110 DAP. There were differences in the proanthocyanidin contents of the ‘TLHL’ and ‘TLHH’ jujube peels during development (Figure 2g), and the proanthocyanidin contents of the ‘TLHL’ jujube peels showed a “double-peak” trend, which increased rapidly to 8.97 mg/g and 9.93 mg/g at the 50 DAP and 95 DAP stages, respectively. The proanthocyanidins content of the ‘TLHH’ jujube peel showed a “unimodal” trend, reaching a maximum of 13.64 mg/g at 80 DAP and then rapidly decreasing to 3.26 mg/g at 110 DAP in the fully red period. The anthocyanin content of the ‘TLHH’ jujube peel was always significantly higher than that of ‘TLHL’ during development (Figure 2h), and its content showed a decreasing trend, with the highest content of 8.11 mg/g at 30 DAP. The anthocyanin content of the ‘TLHL’ jujube peel showed an increasing trend, and no anthocyanin was detected from 30 DAP to 50 DAP; the anthocyanin content reached the highest level (2.31 mg/g) at the 110 DAP fully red stage.

A principal component analysis (PCA) was carried out in the eight pigment substances of the ‘TLHL’ and ‘TLHH’ jujube peels during development (Figure 2i), and it was found that the first three main components could be used to effectively compare the content differences in the of eight pigment substances of the ‘TLHL’ and ‘TLHH’ jujube peels during development. According to the extraction results of the eight pigments from the ‘TLHL’ and ‘TLHH’ jujube peels (see Table A2), further analysis showed that the first principal component of the jujube peel pigment was represented by chlorophyll b, total chlorophyll, and chlorophyll a; the second principal component was represented by carotenoids and total phenols; and the third principal component was represented by anthocyanins. As the cumulative contribution rate of the variance of the first three main components reached 92.76%, the above six substances could effectively reflect the content distribution of the ‘TLHL’ and ‘TLHH’ jujube peel substances. The results indicated that chlorophyll, carotenoids, and anthocyanins were the main substances responsible for the difference in the color of the ‘TLHL’ and ‘TLHH’ jujube peels.

### 3.3. Analysis of Carotenoid Metabolites during Jujube Peel Development

**Figure 3:**
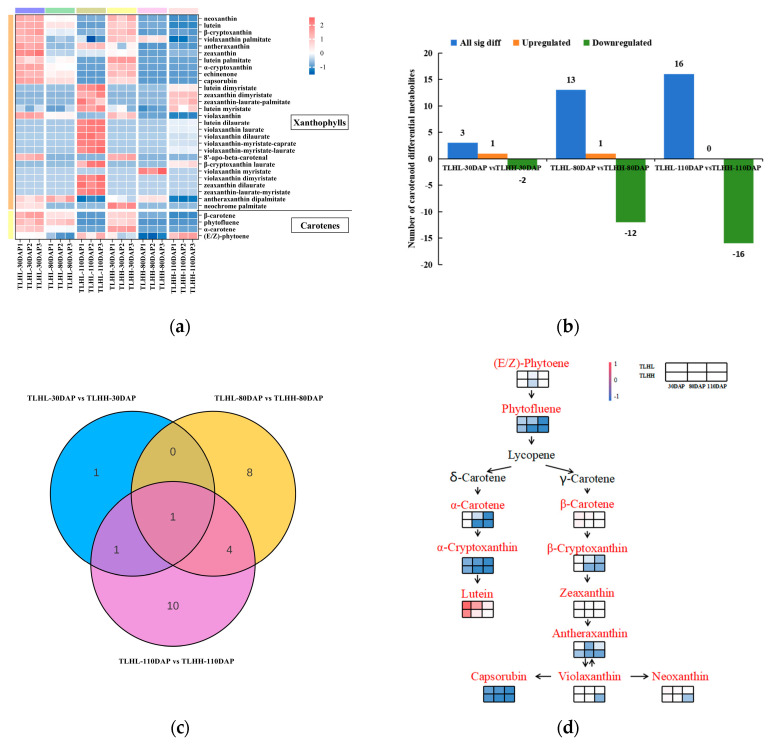
Analysis of carotenoid metabolism in the peel during jujube fruit development. (**a**) Heatmap of carotenoid metabolites clustering in the peel of jujube fruit during fruit development. In a cluster heatmap, each column represents a sample and each row represents a metabolite. Different colors in the cluster heatmap represent different metabolite contents. Red and blue indicate higher or lower levels of metabolites, respectively; (**b**) the number of upregulated and downregulated differential metabolites of carotenoids in the jujube peel. (**c**) Venn diagrams of differential metabolites among the three comparison groups (‘TLHL’-30 DAP vs. ‘TLHH’-30 DAP, ‘TLHL’-80 DAP vs. ‘TLHH’-80 DAP, and ‘TLHL’-110 DAP vs. ‘TLHH’-110 DAP); (**d**) schematic diagram of the carotenoid metabolic pathways and relative levels of carotenoid metabolite in the jujube peel. The red names represent metabolites that were significantly different in the ‘TLHL’ and ‘TLHH’ fruits, while the black names represent compounds that were not detected.

In order to study the composition and dynamic changes in the pigments in the ‘TLHL’ and ‘TLHH’ jujube peels during the color change process, LC-MS/MS was used to metabolomic analyze the key pigment substances causing the color differences between the two color-presenting types of jujube fruits at the key color-transferring stages of 30 DAP, 80 DAP, and 110 DAP. A total of 32 carotenoids were identified in the peels of ‘TLHL’ and ‘TLHH’ (Figure 3a), including 4 carotenoids and 28 xanthophylls, of which lutein was the major carotenoid.

In order to identify the carotenoid differential metabolites in the ‘TLHL’ and ‘TLHH’ jujube peels during development, their relative contents were compared separately. The results showed (Figure 3b) that the comparison between ‘TLHL’-30 DAP and ‘TLHH’-30 DAP revealed differences in the three carotenoid substances, in which the neochrome palmitate compound was upregulated and the antheraxanthin and zeaxanthin compounds were downregulated. The comparison between ‘TLHL’-80 DAP and ‘TLHH’-80 DAP showed that 13 carotenoids were significantly upregulated and downregulated, among which only the violaxanthin myristate compounds were upregulated. The number of differentially expressed carotenoids between ‘TLHL’-110 DAP and ‘TLHH’-110 DAP was 16, and all of them were downregulated. In addition, there was only one common differential metabolite, zeaxanthin, in all three comparison groups (Figure 3c).

A total of 25 carotenoid differential metabolites were detected in the jujube peel samples of ‘TLHL’ and ‘TLHH’ at different coloring stages, of which 12 carotenoid differential metabolites were annotated into 5 metabolic pathways in the KEGG database, including metabolic pathways, carotenoid biosynthesis, biosynthesis of various plant secondary metabolites, biosynthesis of secondary metabolites, and biosynthesis of cofactors. Twelve metabolites, including (E/Z)-phytoene, phytofluene, α-carotene, β-carotene, α-cryptoxanthin, β-cryptoxanthin, lutein, zeaxanthin, antheraxanthin, violaxanthin, neoxanthin, and capsorubin, were enriched in the biosynthesis of the cofactors of the metabolic pathway of carotenoids (Figure 3d), among which the lutein content was the highest, with the content of ‘TLHL’ (93.05 µg/g) reaching a maximum at 30 DAP, which was higher than that of ‘TLHH’ (74.14 µg/g). Both ‘TLHL’ and ‘TLHH’ showed a decreasing trend along with the ripening of the fruits. The results showed that the content of lutein metabolites was negatively correlated with coloring during carotenoid metabolism. It can be seen that the decrease in lutein content indicates fruit ripening and the deposition of jujube peel pigment.

### 3.4. Analysis of Anthocyanin Metabolites during Jujube Peel Development

**Figure 4:**
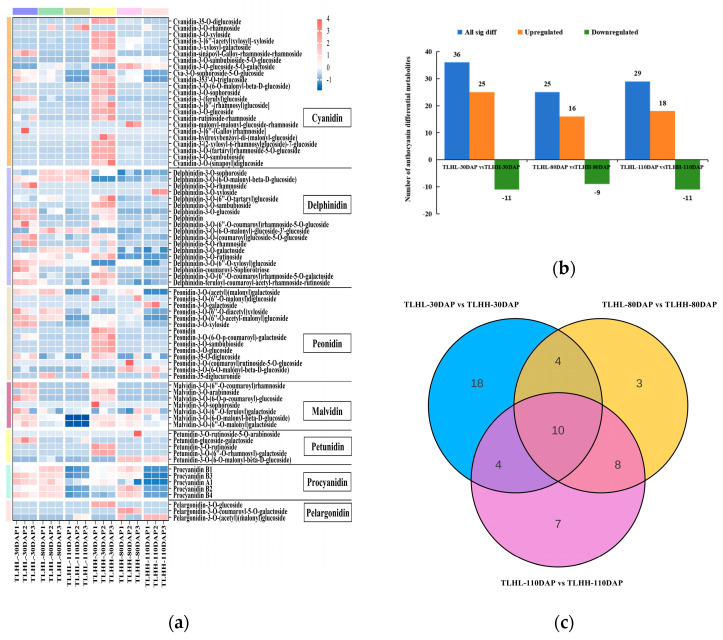

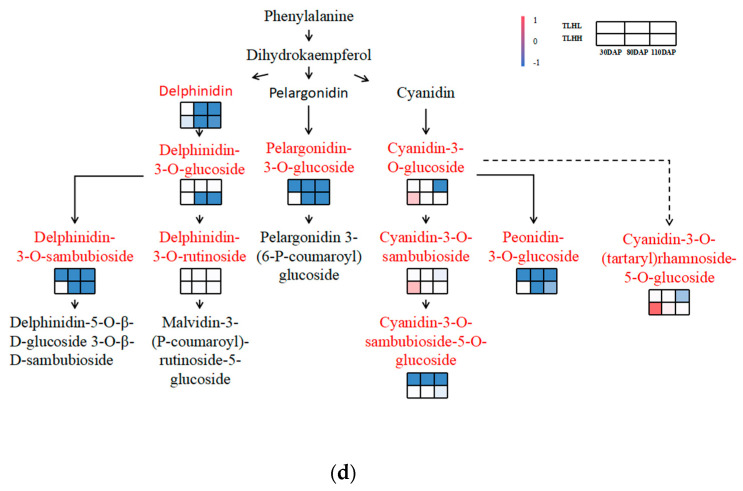
Analysis of anthocyanidin metabolism in the peel during jujube fruit development. (**a**) Heatmap of anthocyanidin metabolites clustering in the peel of jujube fruit during fruit development. In a cluster heatmap, each column represents a sample and each row represents a metabolite. Different colors in the cluster heatmap represent different metabolite contents. Red and blue indicate higher or lower levels of metabolites, respectively; (**b**) the number of upregulated and downregulated differential metabolites of anthocyanidins in the jujube peel. (**c**) Venn diagrams of differential metabolites among the three comparison groups (‘TLHL’-30 DAP vs. ‘TLHH’-30 DAP, ‘TLHL’-80 DAP vs. ‘TLHH’-80 DAP, and ‘TLHL’-110 DAP vs. ‘TLHH’-110 DAP); (**d**) schematic diagram of the anthocyanidin metabolic pathways and relative levels of anthocyanidin metabolite in the jujube peel. The red names represent metabolites that were significantly different in the ‘TLHL’ and ‘TLHH’ fruits, while the black names represent compounds that were not detected.

The anthocyanin metabolism of the jujube peels of ‘TLHL’ and ‘TLHH’ in the 30 DAP, 80 DAP, and 110 DAP periods was analyzed using LC-MS/MS, and a total of 75 anthocyanins were identified (Figure 4a), including 23 cyanidins, 18 delphinidins, 14 peonidins, 5 procyanidins, 7 malvidins, 5 petunidins, and 3 pelargonidins.

In order to identify the anthocyanin differential metabolites in the ‘TLHL’ and ‘TLHH’ jujube peels during development, their relative contents were compared separately. The results showed (Figure 4b) that there were 36, 25, and 29 differential anthocyanin metabolites in the 3 combinations of TLHL’-30 DAP vs. ‘TLHH’-30 DAP, ‘TLHL’-80 DAP vs. ‘TLHH’-80 DAP, and ‘TLHL’-110 DAP vs. ‘TLHH’-110 DAP, respectively, with 25, 16, and 18 upregulated anthocyanin metabolites and 11, 9, and 11 downregulated anthocyanin metabolites in each combination, respectively. In addition, there were 10 common differential metabolites in all 3 comparison groups (Figure 4c), among which the content of cyanidin-3-O-(tartaryl)rhamnoside-5-O-glucoside reached a maximum at 30 DAP in ‘TLHH’ (258.32 µg/g), which was 51.6 times higher than that of ‘TLHL’, and both of them showed a decreasing trend with fruit ripening. It is suggested that cyanidin-3-O-(tartaryl)rhamnoside-5-O-glucoside may play an important role in the anthocyanin synthesis pathway, especially at 30 DAP, where cyanidin-3-O-(tartaryl)rhamnoside-5-O-glucoside is the key metabolite for the coloring of the ‘TLHH’ jujube peel to a purplish red.

A total of 54 anthocyanin differential metabolites were detected in the jujube peel samples of ‘TLHL’ and ‘TLHH’ at the different coloring stages; of these, 15 anthocyanin differential metabolites were annotated into four metabolic pathways in the KEGG database, including metabolic pathways, biosynthesis of secondary metabolites, flavonoid biosynthesis, and anthocyanin biosynthesis. Among them, the contents of cyanidin-3-O-sambubioside and cyanidin-3-O-glucoside were higher in the anthocyanin biosynthesis metabolic pathway (Figure 4d), especially in the ‘TLHH’ peel at 30 DAP, where they were 115.63 µg/g and 93.70 µg/g, respectively. It is shown that in the anthocyanin metabolic pathway, the above two differential metabolites synergized with cyanidin-3-O-(tartaryl)rhamnoside-5-O-glucoside together to participate in the coloring of the ‘TLHH’ jujube peel to a purplish red. In addition, we also found that the content of delphinidin-3-O-(6-O-malonyl-beta-D-glucoside) increased with the ripening and coloring of jujube fruits, but this metabolite was not enriched in the anthocyanin metabolism pathway, and delphinidin-3-O-glucoside in the anthocyanin metabolism pathway had a trade-off relationship with it. It can be seen that delphinidin-3-O-glucoside is indirectly involved in the coloring of jujube fruits at the ripening stage.

### 3.5. Correlation Analysis of Key Differential Metabolites of Carotenoids and Anthocyanins in the Peel during Jujube Development

**Figure 5:**
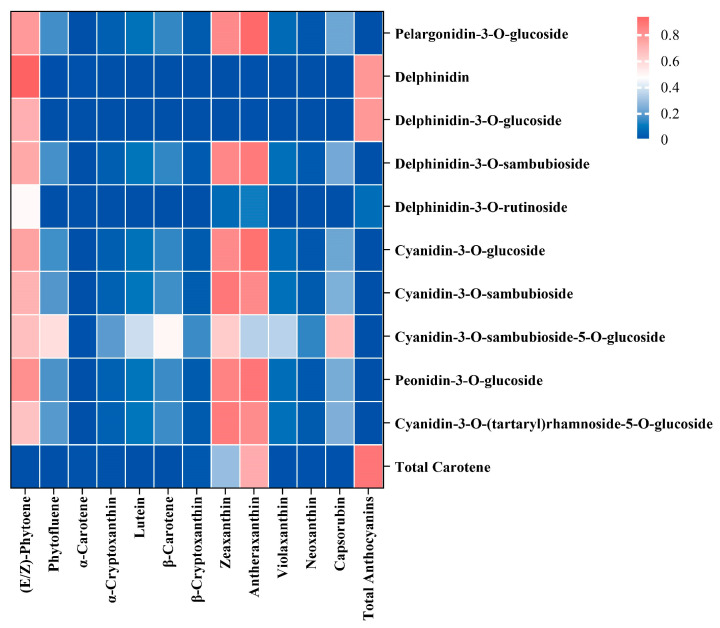
Correlation heatmap of key differential metabolites of peel carotenoids and anthocyanins during jujube fruit development. *p* < 0.05 indicates a significant difference, and *p* < 0.01 indicates a very significant difference.

In order to further explain the difference in the color of the ‘TLHL’ and ‘TLHH’ jujube peels, a metabolic association was made between anthocyanins and carotenoids, which are closely related to the color of jujube peels (Figure 5). In the association analysis of the carotenoid metabolites, only (E/Z)-phytoene was significantly positively correlated with the total carotenoids during the coloring process of the ‘TLHL’ and ‘TLHH’ jujube fruits; lutein with the highest content was significantly negatively correlated with the total carotenoids, and lutein was significantly positively correlated with the other 11 carotene metabolites. In the correlation analysis of the anthocyanin metabolites, there was a positive correlation between all anthocyanin metabolites, and there was a significant correlation between cyanidin-3-O-(tartaryl)rhamnoside-5-O-glucoside, cyanidin-3-O-sambudiglycoside, and cyanidin-3-O-glucoside metabolites with high content; the three substances were significantly positively correlated with the total anthocyanins. Between the anthocyanin and carotenoid metabolites, lutein showed highly significant positive correlations only with delphinidin, delphinidin-3-O-glucoside, and delphinidin-3-O-rutinoside. There was a significant positive correlation between cyanidin-3-O-(tartaryl)rhamnoside-5-O-glucoside, cyanidin-3-O-sambudiglycoside, and cyanidin-3-O-glucoside and α-carotene, α-cryptoxanthin, β-cryptoxanthin, and neoxanthin. These results indicated that α-carotene, α-cryptoxanthin, β-cryptoxanthin, neoxanthin, delphinin-3-O-glucoside, and delphinin-3-O-rutinoside were involved together in the coloring of the ‘TLHL’ and ‘TLHH’ jujube fruits.

## 4. Discussion

The color of the fruit is one of the most important factors influencing consumer choice, and it determines the commercial value of the fruit. In this study, we determined the content changes in a large number of pigment substances, including fat-soluble and water-soluble pigments, during the coloring process of the fruit peels of the ‘TLHL’ and ‘TLHH’ jujube fruits. This was conducted in order to better understand the dynamic changes and coloring differences of different types of jujube fruits and to lay a theoretical foundation for further research on jujube peel coloring.

Fruit ripening is accompanied by chlorophyll degradation and carotenoid accumulation, which in turn leads to peel color change [24,25]. In this study, the contents of chlorophyll a, b, and total chlorophyll in ‘TLHL’ and ‘TLHH’ jujube fruits showed a decreasing trend. The carotenoids showed an increasing trend during fruit development, and the chlorophyll content of the ‘TLHL’ jujube peel was significantly higher than that of ‘TLHH’; thus, it is assumed that chlorophyll plays an important role in the green coloration of ‘TLHL’ jujube fruits compared with ‘TLHH’. Shi et al. [9] also demonstrated that carotenoids showed an upward trend with the decrease in chlorophyll during the development of jujube, indicating that carotenoids were involved in fruit coloration in the late stage. Therefore, it is assumed that there is a trade-off relationship between peel chlorophyll and carotene during fruit development. The contents of flavonoids, total phenols, proanthocyanidins, and anthocyanins in jujube fruit have shown different trends in different studies. Li [8] found that the flavonoid content of the peel of the ‘Dongzao’ jujube fruit decreased during the development of the fruit and that the content of proanthocyanidins increased first and then decreased; meanwhile, Shi’s [9] study showed that the flavonoid content of the peel of the ‘Junzao’ jujube increased first and then decreased and that the anthocyanins remained at a low level. In this study, the contents of flavonoids, total phenols, and proanthocyanidins in the peels of the ‘TLHL’ and ‘TLHH’ jujube fruits showed, as a whole, an increasing and then decreasing trend. These results indicated that the increase in flavonoids, total phenols, proanthocyanidins, and other pigment substances in the peel of ripe jujube marked the beginning of pigment synthesis and decreased the marker pigment deposition; these results also suggested that the increase in the contents of flavonoids, total phenols, and proanthocyanidins in the peel of ripe jujube marked the beginning of pigment synthesis and decreased the pigment deposition. Conversely, the anthocyanin content of ‘TLHH’ was highest at the young fruit stage and showed a decreasing and then increasing trend along with the ripening of the fruit, while ‘TLHL’ had the highest content only at the fully red stage. You [26] found that the anthocyanin content of ‘Dongzao’ jujube peel was the highest at the fully red stage, and its change rule was consistent with that of ‘TLHL’ in this study. Li’s [8] research results showed that the anthocyanin content of the peel of ‘Dongzao’ jujube increased and then decreased with the coloring of the fruit and that its content was highest at the semi-red stage. This indicated that there were differences in the pigment content of different varieties along with the development of peel color.

Carotenoids are widely found in plants and are important organic pigments for the yellow, orange, and red colors of fruits [27], and their accumulation has been studied in some depth in fruit trees such as apricots, bananas, and peaches [28,29,30,31]. Research on the pigmentation of jujube started late, and few studies have been reported on the relationship between jujube peel carotenoids and color presentation. In this study, carotenoid metabolites were analyzed for the first time in jujube peel using LC-MS/MS; a total of 32 carotenoids were identified in the peels of ‘TLHL’ and ‘TLHH’ at the three key color change stages, including 4 carotene compounds and 28 xanthophyll compounds, among which lutein was the main carotenoid substance, and its content was about 10 times higher than that of β-carotene at the young fruit stage. In contrast, Shi [9] showed that the β-carotene content of the peel of ‘Junzao’ and ‘TLHH’ jujube was 3–4 times higher than that of lutein at the young fruit stage. These results indicated that although ‘TLHL’ and ‘TLHH’ were derived from the same maternal parent, there were significant differences in the main components of carotenoids compared with the maternal parent. Furthermore, in the results of a study by Shi [9], it was found that lutein showed a decreasing and then increasing trend, and its pattern of change seems to be similar to the color change in the peel of jujube fruit. The results of this study were different from those in the above in that ‘TLHL’ and ‘TLHH’ jujube peel lutein showed a decreasing trend; however, the change trend of (E/Z)-phytoene was similar to the color pattern of the peel, and there was a significant positive correlation with total carotenoids. Therefore, it is assumed that (E/Z)-phytoene may be involved in the color difference of the peel in the carotenoid metabolism pathway.

Anthocyanin is a red-blue water-soluble pigment with a red to purple color range; it is commonly found in plants as cyanidin, delphinidin, pelargonidin, peonidin, petunidin, and malvidin [32]. Shi [9] identified a total of 171 flavonoids in the peel of ‘Junzao’ and ‘Tailihong’ jujube at different developmental stages. Among these flavonoids, 13 anthocyanins were cyanidins, of which cyanidin-3-glucoside, cyanidin-3-rutinoside, cyanidin, and paeonidinin 3,5-O glucoside were the main components, in the anthocyanins of jujube peel. In this study, a total of 75 anthocyanins were identified using LC-MS/MS on the peels of ‘TLHL’ and ‘TLHH’ jujube at three stages, including 23 cyanidins, 18 delphinins, 14 paeoniflorins, 5 proanthocyanidins, 7 mallows, 5 petunidins, and 3 pelargonoids. Among them, cyanidin-3-O-(tartaryl)rhamnoside-5-O-glucoside, cyanidin-3-O-sambubioside, and cyanidin-3-O-glucoside might be the key substances leading to the difference in color between ‘TLHL’ and ‘TLHH’ jujube peels at the young fruit stage, and the content of cyanidin-3-O-(tartaryl)rhamnoside-5-O-glucoside was the highest. With the ripening of the fruits, the color of the peels of ‘TLHL’ and ‘TLHH’ was almost the same, but there were still significant differences in the anthocyanin contents, and the content of delphinin was higher, suggesting that delphinidins may be involved in the color of the peel of jujube fruits when they are ripe. However, Zhang [10], using ‘Dongzao’ jujube as the test material, showed that a total of five flavonols and eight flavanols were detected in the peel during the coloring process, and no anthocyanosides were detected. Later, Zhang [7] detected 13 anthocyanins in jujube peel and speculated that delphinidin, delphinin 3-O glucoside, and dimethylsphinin 3-O glucoside might be the key anthocyanins that cause the coloring of jujube peel. Thus, it is clear that the anthocyanin components involved in fruit color need to be further verified when the jujube fruit is ripe.

In this study, it was confirmed that lutein, cyanidin-3-O-(tartaryl)rhamnoside-5-O-glucoside, cyanidin-3-O-sambudiglycoside, and cyanidin-3-O-glucoside were the main metabolites responsible for the color difference between ‘TLHL’ and ‘TLHH’, which provides a theoretical basis for the improvement of the appearance quality of jujube fruits and has broad application prospects and important significance for the cultivation of jujube germplasm with excellent commercial traits. However, in the process of color change of ‘TLHL’ and ‘TLHH’, other secondary colored metabolites are still unclear, and their potential utilization value in the future remains to be explored.

## 5. Conclusions

In this study, 30 DAP, 80 DAP, and 110 DAP were the key periods for the difference in color between the ‘TLHL’ and ‘TLHH’ jujube peels. The main differentiating substances at the early stage of the peel were chlorophyll, carotenoids, and anthocyanins, and the color of both fruits tended to be the same as the fruits ripened and developed. Furthermore an LC-MS/MS metabolic analysis showed that lutein, cyanidin-3-O-(tartaryl)rhamnoside-5-O-glucoside, cyanidin-3-O-sambudiglycoside, and cyanidin-3-O-glucoside were the key metabolites responsible for the color difference between ‘TLHL’ and ‘TLHH’ and were mainly expressed at 30 DAP. At the same time, the metabolic correlation analysis between the metabolites closely related to the coloration of jujube peel showed that α-carotene, α-cryptoxanthin, β-cryptoxanthin, neoxanthin, delphinin-3-O-glucoside, delphinin-3-O-rutinoside, and other metabolites were collectively involved in the coloring of ‘TLHL’ and ‘TLHH’ jujube fruits.

## Data Availability

The original contributions presented in the study are included in the article, further inquiries can be directed to the corresponding author.

## Appendix A

**Table A1:** Regression equation for the standard of jujube peel pigment substances.

Substances/Standards	Standard Curve Regression Equation	R^2^
Total phenols (mg GAE/g)	Y = 0.01193 X + 0.00007	0.9996
Flavone (mg RE/g)	Y = 0.0383 X − 0.0007	0.9993
Proanthocyanidins (mg CE/g)	Y = 0.0513 X + 0.0096	0.9954

**Table A2:** Extraction results for the principal components of jujube peel pigments.

Pigmented Substance	Ingredient 1	Ingredient 2	Ingredient 3
Chlorophyll a	0.917	0.381	0.044
Chlorophyll b	0.934	0.269	0.192
Total chlorophyll	0.929	0.355	0.051
Carotenoids	−0.256	−0.896	0.055
Flavonoids	−0.532	0.648	−0.396
Total Phenols	−0.464	0.816	0.002
Proanthocyanidins	−0.553	0.744	0.217
Anthocyanins	−0.386	0.064	0.888
Eigenvalue	3.595	2.789	1.037
Percentage of variance %	44.932	34.86	12.965
Cumulative percentage %	44.932	79.792	92.757

## References

[1] Li X., Wang T.T., Zhou B., Gao W.Y., Cao J.G., Huang L.Q. (2014). Chemical compositionand antioxidant and anti-inflammatory potential of peels and flesh from 10 different pear varieties (*Pyrus* spp.). Food Chem..

[2] Lee K.H., Cho J.Y., Le H.J., Ma Y.K., Kwon J., Park S.H., Lee S.H., Cho J.A., Kim W.S., Park S.H. (2011). Hydroxycinnamoylmalic acids and their methyl esters from pear (*Pyrus pyrifolia* Nakai) fruit peel. J. Agric. Food Chem..

[3] Scordino M., Sabatino L., Muratore A., Belligno A., Gagliano G. (2012). Phenolic Characterization of Sicilian Yellow Flesh Peach(*Pruous persicu* L.) Cultivars at Different Ripening Stages. J. Food Qual..

[4] Yan J., Shen Z.J., Cai Z.X., Yu M.L. (2014). Advances of study on phenolic compounds in peach fruit. J. Fruit Sci..

[5] Wu C.S., Gao Q.H., Guo X.D., Yu J.G., Wang M. (2012). Effect ofripening stage on physicochemical properties and antioxidant profilesof a promising table fruit ‘pear-jujube’ (*Zizyphus jujuba* Mill.). Sci. Hortic..

[6] Sun S., Xin L., Gao H.J., Wang J.Y., Li P.M. (2014). Response of phenolic compounds in ‘Golden Delicious’ and ‘Red Delicious’ apples peel to fruit bagging and subsequent sunlight re-exposure. Sci. Hortic..

[7] Zhang Q. (2020). Analysis of Peel Structure and Components Related to Pigment Accumulation during Jujube Coloring. Ph.D. Thesis.

[8] Li Y.F. (2017). The Molecular Mechanism Research on the Red Pigment Development of *Zizyphus Jujuba* Mill cv. Dongzao. Master’s Thesis.

[9] Shi Q.Q. (2019). Molecuar Mechanism of the Formation of Fruit Pigment in Jujube Fruits. Ph.D. Thesis.

[10] Zhang Q., Zhou G.F., Shen G.N., Zhu E.Y., Wang H.Q. (2010). The flavonoids in the fruit peel of *Ziziphus jujuba* Mill. ’Dongzao’ during coloring process. Acta Hortic. Sin..

[11] Cai L.P. (2020). Mechanism of Jujube Reddening Based on Transcriptome Sequencing. Master’s Thesis.

[12] Xia D.L. (2006). The Studies on the Stability and Qualitative and Quantitative Analysis of Flavonoids in Pigment of Winter Jujube Peel. Master’s Thesis.

[13] Xu H.F., Wang Z.T., Chen X., Liu Z.G., Wang L.H., Liu P., Liu M.J., Zhang Q. (2022). The analyses of target metabolomics in flavonoid and its potential MYB regulation factors during coloring period of Winter Jujube. Acta Hortic. Sin..

[14] Li X., Shi Q.Q., Zhu D.J., Du J.T., Li X.G. (2020). The patterns of flavonoids accumulation and the expression of biosynthesis related genes during the course of maturation of the Chinese jujube fruit. J. Fruit Sci..

[15] Li W.Y., Wang Z., Yuan Q.F., Chen S.Y., Li J.Q. (2013). Fruit qualities and carotenoid contents of niurouhong mandarin from different areas. Southwest China J. Agric. Sci..

[16] Jiang X., Tang Z.H., Wu C.Y., Wang X., Pu Y.F., Guo L. (2021). Phenolic composition and antioxidant capacity of developing pear fruit from three cultivars. Food Sci..

[17] Chen Q. (2015). Comprehensive Evaluation of the Main Functional Factors and Identification of the Antioxidant and Tumor-Inhibition Compounds in Jujube. Master’s Thesis.

[18] Huang D., Wang X., Tang Z.Z., Yuan Y., Xu Y.T., He J.X., Jiang X.L., Peng S.A., Li L., Butelli E. (2018). Subfunctionalization of the *Ruby2-Ruby1* gene cluster during the domestication of citrus. Nat. Plants.

[19] Amorim-Carrilho K.T., Cepeda A., Fente C., Regal P. (2014). Review of methods for analysis of carotenoids. Trends Anal. Chem..

[20] Krinsky N.I., Mayne S.T., Sies H. (2004). Carotenoids in Health and Disease.

[21] Geyer R., Peacock A.D., White D.C., Lytle C., Van G.J. (2004). Atmospheric pressure chemicalionization and atmospheric pressure photoionization forsimultaneous mass spectrometric analysis of microbial respiratory ubiquinones and menaquinones. J. Mass Spectrom..

[22] Cruz A.A.D.L., Hilbert G., Rivière C., Mengin V., Ollat N., Bordenave L., Decroocq S., Delaunay J.C., Delrot S., Mérillon J.M. (2012). Anthocyanin identification and composition of wild *Vitis* spp. accessions by using LC-MS and LC-NMR. Anal. Chim. Acta.

[23] Ferrars R.M.D., Czank C., Saha S., Needs P.W., Zhang Q.Z., Raheem K.S., Botting N.P., Kroon P.A., Kay C.D. (2014). Methods for Isolating, Identifying, and Quantifying Anthocyanin Metabolites in Clinical Samples. Anal. Chem..

[24] Hu Y.Y., Wang G.H., Pan S.Y., Wang L.F. (2018). Influence of ethylene and ethephon treatments on the peel color and carotenoids of Gannan Newhall navel orange during postharvest storage. J. Food Biochem..

[25] Peng G., Xie X.L., Jiang Q., Song S., Xu C.J. (2013). Chlorophyll a/b binding protein plays a key role in natural and ethylene-induced degreening of Ponkan (*Citrus reticulata* Blanco). Sci. Hortic..

[26] You F., Huang L.X., Zhang C.H., XIE P.J., Zhang Y.L. (2013). Preliminary study on spectrums and structure properties of pigments from *Ziziphus* jujube peel. Food Ind. Sci. Technol..

[27] Sagawa J.M., Stanley L.E., LaFountain A.M., Frank H.A., Liu C., Yuan Y.W. (2016). An R2R3-MYB transcription factor regulates carotenoid pigmentation in *Mimulus lewisii* flowers. New Phytol..

[28] Marty I., Bureau S., Sarkissian G., Gouble B., Audergon J.M., Albagnac G. (2005). Ethylene regulation of carotenoid accumulation and carotenogenic gene expression in colour-contrasted apricot varieties (*Prunus armeniaca*). J. Exp. Bot..

[29] Ayour J., Sagar M., Alfeddy M.N., Taourirte M., Benichou M. (2016). Evolution of pigments and their relationship with skin color based on ripening in fruits of different Moroccan genotypes of apricots (*Prunus armeniaca* L.). Sci. Hortic..

[30] Heng Z., Sheng O., Huang W., Zhang S., Fernie A.R., Motorykin L., Kong Q., Yi G.J., Yan S.J. (2019). Integrated proteomic and metabolomic analysis suggests high rates of glycolysis are likely required to support high carotenoid accumulation in banana pulp. Food Chem..

[31] Cao S.F., Liang M.H., Shi L.Y., Shao J.R., Song C.B., Bian K., Chen W., Yang Z.F. (2017). Accumulation of carotenoids and expression of carotenogenic genes in peach fruit. Food Chem..

[32] He J., Giusti M.M. (2010). Anthocyanins: Natural colorants with health-promoting properties. Annu. Rev. Food Sci. Technol..

